# Editorial: Insights in allergology: 2021/22

**DOI:** 10.3389/falgy.2023.1223904

**Published:** 2023-06-14

**Authors:** Gabriele Gadermaier, Birgit Linhart, Ines Swoboda

**Affiliations:** ^1^ Biosciences and Medical Biology, Paris Lodron University Salzburg, Salzburg, Austria; ^2^ Department of Pathophysiology and Allergy Research, Medical University of Vienna, Vienna, Austria; ^3^ Molecular Biotechnology Section, FH Campus Wien, University of Applied Sciences, Vienna, Austria

**Keywords:** geographic sensitization patterns, innate immune responses to allergens, allergen families, epitope mapping, allergen silencing

**Editorial on the Research Topic** Insights in allergology: 2021/22

Knowledge on allergens, the molecular elicitors of Type I allergic reactions, is key to the broad understanding of IgE-mediated allergic responses. Thus, the ‘Allergens’ section of *Frontiers in Allergy* launched the Research Topic *Insights in Allergology: 2021/22* that compiles four review articles, one perspective, and one original research article on new, interesting aspects, all centered around the topic of allergens, such as geographic sensitization patterns, innate immune responses to allergens, allergen families, epitope mapping, and allergen silencing in allergen sources.

Considered for a long time as a problem of the westernized world, allergies also seem to be on the rise in Sub-Saharan Africa, but accurate data are still lacking and need to be translated into practice (Mvoundza Ndjindji and Djoba Siawaya). Patients are frequently polysensitized and predominately in the context of asthma, mites, cockroaches, molds, and pet dander were identified as relevant allergen sources. It is estimated that 5%–50% of allergic reactions in Africa are caused by foods. However, food allergy lacks systematic documentation and often relies solely on self-reported symptoms or skin-prick-tests, limiting data reliability. Precise allergy testing requires both, a clinical diagnosis as well as confirmatory allergen-specific reaction. Therefore, Mvoundza Ndjindji et al. suggest stringent and accurate diagnosis to improve allergy mapping in Africa. This could allow the introduction of precision medicine, which offers patient-tailored treatment approaches, including specific allergen avoidance.

While the adaptive immune response to allergens is characterized by the development of Th2 cells and allergen-specific IgE antibodies, there is a growing body of literature focusing on the contribution of innate immune cells to the initiation and perpetuation of allergic disease (1). It becomes more and more evident that some allergen molecules themselves could activate type 2 innate immunity and act as their own adjuvants. Since the major house dust mite allergen Der p 2 was identified as a structural and functional homologue of MD-2, the LPS-binding component of the Toll-like receptor 4 signaling complex, additional reports on the interaction of allergens with the innate immune system followed (2). A review by Virtanen highlights the role of lipocalin allergens in this context. As the lipocalin protein family represents a major part of mammalian respiratory allergens (e.g., from cat and dog), it could be speculated that these allergens promote their own allergenicity. The author critically discusses possible intrinsic adjuvant effects of lipocalins, like the specific cell-surface receptor binding of lipocalins favoring their active uptake and facilitating access to the immune system. However, also extrinsic adjuvant effects by co-delivered immunomodulatory components like LPS are considered. Roth-Walter also takes a closer look at the role of allergens in innate immune priming in her review article, however from another perspective. Specifically, the ability of some allergens to bind ligands representing important micronutrients in humans, such as iron complexes, flavonoids, and vitamins, and manipulating the immune system via the bioavailability of these substances are discussed.

Features that contribute to allergenicity are also addressed in the case of a recently identified family of allergens, namely the gibberellin-regulated proteins (GRPs). This plant protein family was shown to contain allergenic as well as non-allergenic molecules (3). Therefore, GRPs might represent a valuable model system for gaining a better understanding about parameters that contribute to the allergenicity of proteins. In their recent perspective Iizuka et al. summarize the current knowledge on this plant allergen family. They describe the GRPs as small, basic proteins, stabilized by disulfide bridges, which confer resistance to heat and proteolytic treatment. The authors further mention the IgE cross-reactivity between fruit GRPs and Cupressaceae pollen GRPs, which seems to be the basis for the observed citrus/cypress and peach/cypress allergy syndromes. Iizuka et al. speculate why, despite their wide distribution in the plant kingdom and their conserved amino acid sequences, allergenic GRPs have only been identified in nine plant species. GRPs might require cofactors, such as physical exercise, alcohol or stress, or the binding of specific ligands for allergic sensitization and for triggering allergic symptoms.

Key to understanding disease- and treatment-related responses during allergen-specific immunotherapy (AIT) is the knowledge of IgE and IgG epitopes (4). Although most epitopes of inhalant allergens are conformational, linear epitopes can provide additional insights, especially with respect to therapeutic IgG antibodies. The advantage of studying linear epitopes is that they are easily synthesized and evaluated in high-throughput arrays. Thörnqvist et al. focused on group 5 and minor grass pollen allergens and using array technology they analyzed the IgE and IgG reactivity to 16-mer peptides in patients undergoing grass pollen immunotherapy and control patients. A unique and high-resolution feature of this work is the representation of protein sequences from all allergenic grass species, including isoallergens and variants. IgE reactivity to linear peptides was limited and not further induced during AIT. Before treatment, the number of recognized IgG epitopes was similar in all patients while the number of IgG-reactive peptides increased significantly only during AIT. The location of the epitopes was mostly patient-specific and remained stable over the 3-year study period regardless of treatment. An exception was one Phl p 5.0101 epitope, which was induced in 4/5 AIT patients, while none of the controls reacted to this IgG epitope. Further analysis comparing isoallergenic peptides suggests that this region contains two overlapping but independent epitopes, both located in the flexible linker region of Phl p 5.

AIT has shown effectiveness and safety for treatment of certain allergies, but for most allergic diseases, allergen avoidance and medications for relief of symptoms are still the preferred management options. In practice, avoidance of allergen exposure is not always easily achieved. Silencing or deletion of allergen encoding genes in their respective source provides an effective way to enable avoidance. Allergen gene silencing strategies can operate at the mRNA level, and reduction of allergen expression can be achieved by antisense approaches (5) or by RNA interference (RNAi) (6). Alternatively, they can operate at the DNA level, and allergen gene knockout can be obtained by genome editing techniques using zinc-finger nucleases (7) or transcription activator-like effector nucleases (8). However, the most powerful technology for genome editing is the CRISPR (Clustered regularly interspaced short palindromic repeats) gene-editing technology. In their review, Brackett et al. focused on the potential of CRISPR editing to knock-out allergen genes and in this way produce hypoallergenic allergen sources. The authors explain the technology and describe the advantages of CRISPR, such as target specificity, ease of use, editing efficiency and precision, but mention also an important disadvantage, the off-target editing. They further describe first promising approaches that showed the applicability of CRISPR editing technology for development of hypoallergenic food (e.g., peanut, soybean, wheat, eggs) and allergen-free animals (e.g., cats). CRISPR genome editing certainly has the potential to revolutionize the management and treatment of allergic diseases. However, it will be of utmost importance that the normal biological functions of the edited allergen sources are retained.

This brief summary shows that the Research Topic *Insights in Allergology 2021/22* offers an inspiring and informative article collection.

## References

[1] Salazar F Ghaemmaghami AM. Allergen recognition by innate immune cells: critical role of dendritic and epithelial cells. Front Immunol. (2013) 4:356. 10.3389/fimmu.2013.00356 24204367 PMC3816228

[2] Trompette A Divanovic S Visintin A Blanchard C Hegde RS Madan R Allergenicity resulting from functional mimicry of a Toll-like receptor complex protein. Nature. (2009) 457(7229):585–8. 10.1038/nature07548 19060881 PMC2843411

[3] Tuppo L Alessandri C Pomponi D Picone D Tamburrini M Ferrara R Peamaclein–a new peach allergenic protein: similarities, differences and misleading features compared to Pru p 3. Clin Exp Allergy. (2013) 43(1):128–40. 10.1111/cea.12028 23278887

[4] Pomes A Mueller GA Chruszcz M. Structural aspects of the allergen-antibody interaction. Front Immunol. (2020) 11:2067. 10.3389/fimmu.2020.02067 32983155 PMC7492603

[5] Bhalla PL Swoboda I Singh MB. Antisense-mediated silencing of a gene encoding a major ryegrass pollen allergen. Proc Natl Acad Sci U S A. (1999) 96(20):11676–80. 10.1073/pnas.96.20.11676 10500236 PMC18093

[6] Dodo HW Konan KN Chen FC Egnin M Viquez OM. Alleviating peanut allergy using genetic engineering: the silencing of the immunodominant allergen Ara h 2 leads to its significant reduction and a decrease in peanut allergenicity. Plant Biotechnol J. (2008) 6(2):135–45. 10.1111/j.1467-7652.2007.00292.x 17784907

[7] Sun Z Wang M Han S Ma S Zou Z Ding F Production of hypoallergenic milk from DNA-free beta-lactoglobulin (BLG) gene knockout cow using zinc-finger nucleases mRNA. Sci Rep. (2018) 8(1):15430. 10.1038/s41598-018-32024-x 30337546 PMC6194018

[8] Cui C Song Y Liu J Ge H Li Q Huang H Gene targeting by TALEN-induced homologous recombination in goats directs production of beta-lactoglobulin-free, high-human lactoferrin milk. Sci Rep. (2015) 5:10482. 10.1038/srep10482 25994151 PMC5386245

